# Special Analytical and Health Commission on Tinned and Preserved Food

**Published:** 1906-08-18

**Authors:** 


### SPECIAL ANALYTICAL AND HEALTH COMMISSION ON TINNED AND PRESERVED FOOD.

### VII. HOGS', BOARS', AND CALVES' HEADS.

Brawn is a dish much in favour with a certain section of the public, and if we inquire carefully into its nature and method of preparation we shall come to the conclusion that it merits its place in dietetics. Brawn is, however, pig's flesh, and pig's flesh of all flesh is the one which has most frequently been the cause of poisoning. Indeed, brawn itself has not a perfectly clean history in this respect, for an epidemic of meat poisoning occurred some time ago at Retford, no fewer than eighty persons being attacked. In this case the meat was pork, and it was partially consumed as pork pies and partially as brawn. One of the peculiarities of pigs' meat is that it furnishes, when appropriately treated, a large proportion of gelatine, and it is, thanks to this gelatine, that brawn can be made. Further, by means of spicing and flavouring this gelatine we get a most pleasant adjuvant to the digestion of the corresponding meat. This jelly, however, has its disadvantages, the chief of which, perhaps, is that it forms an excellent medium for the growth of bacteria; hence it is most important that it should be sufficiently cooked in the first instance to render it absolutely sterile, and that it should be put up in a way adequate to prevent all reinfection. The effect of bacterial growth in or on gelatine is, as a rule, to liquefy it; hence a brawn or a boar's head of which the jelly is not quite solid and clear should be regarded *à priori* with suspicion. Brawn is made, as probably our readers know, from hog's head, which, in the first instance, is pickled much in the way as hams are pickled, an essential constituent of the pickle being saltpetre. After several processes, the details of which we need not describe, the meat is cut into small cubes, treated with the stock, to which, as a rule, spices are added, and pressed firmly into a brawn tin, and allowed to cool. The pleasant red colour of the meat of brawn should be due to the action of the saltpetre upon the hæmoglobin of the flesh. But it was found that in fifty-six samples examined six had their colour reinforced by a very small quantity of a coal-tar dye. If brawn is properly made and hermetically sealed in glass or in an ordinary can it should keep indefinitely, but if after being opened it is exposed, as is very often the case in the larders of the poor, to dirty surroundings, it goes bad fairly rapidly, especially if no preservative has been added. The commoner classes of brawn frequently do contain some chemical preservative, and the one almost invariably used is boracic acid. Of the samples examined by the Departmental Committee on Food Preservatives, 57 per cent. contained this substance. The Committee, however, in their report seemed to regard its use in this class of food as unobjectionable. Pig's head, and hence brawn, is not quite so cheap as it seems—or, more correctly, not quite the amount of profit accrues—to the maker as would at first sight appear to be the case. Only about 60 per cent. of the pig's head can be utilised for eating purposes; the flesh, however, that is utilised is relatively nutritious, and contains a relatively small amount of water—namely, 45 per cent.—and a very large amount of fat—namely, 41 per cent.

We have received from Messrs. Watling and Sons a sample of their Oxford brawn put up in glass. This article is ready for table use, and is of excellent quality. It is obviously prepared from carefully selected hogs' heads; the jelly is firm, clear, and of good flavour. The preparation keeps well after it is opened. We do not purpose describing at length "collared boar's head," of which we have received a sample from Messrs. Chivers and Sons, Limited. Collared boar's head is prepared in a manner similar to brawn, except that the head of the boar is used instead of the head of the hog. Messrs. Chivers' boar's head is put up in glass with pistachia kernels, and also, we notice, the appearance of the preparation has been greatly improved by the fact that its top (when turned out) is formed by a delicately cut piece of tongue. Messrs. Chivers and Sons' collared boar's head is a most delicate and appetising preparation, consisting of sound materials thoroughly well prepared. The same firm have also sent us a specimen of their galantine of chicken, ham, and tongue, which is well up to the high standard of the other products of this maker.

Calves' head has long been known and used as a light and nutritious meat dish. Unfortunately its delicacy (in which, as a matter of fact, from the point of view of dietetics, most of its value consists) depends largely upon the method of its preparation, and the most exhaustive resources of the culinary art have been employed to render this dish attractive and appetising. It consists essentially of the muscular flesh of the muscles of the face, the brains, and the salivary glands, and each helping should contain a portion of these constituents. It is rich in fat, and its fat consists of the loose adipose tissue of the calf's neck. Perhaps, however, its most distinctive feature is the extreme tenderness and digestibility of its nutritive elements. Of the culinary adjuncts, since they are so varied, we can say little; but they are, for the most part, employed as appetisers and for the purpose of increasing the secretion of the digestive juices. We have received from Messrs. P. Winter and Company a sample of their Tête de Veau en Tortue. This preparation is, of course, intended to be eaten hot, and directions are given on the tin as to how it should be prepared for table. The taste and appearance of Messrs. Winter's calves' head are excellent, and we regard it as a delicate and valuable accessory to any larder. The preparation of calves' head, if this is done *ab initio* at home, is both costly and complicated, and hence we regard this as one of the dietetic preparations which lend themselves with the greatest justification to the preservers' art.

### Potted Meats and Pates.

The exigencies of modern life have forced upon mankind the habit of eating in a hurry. Teeth have not risen to the occasion, and, as far as we know, are worse now than when the human race had the time to eat at leisure. Whatever doctors and philosophers may say to the contrary, the average man, even if he has thirty-two teeth to bite with, hardly bites at all, but leaves food in his mouth for just sufficient time to taste it, and then swallows it, forgetting that he has neither a gizzard like a bird nor teeth in his stomach like a lobster, and that the mechanical preparation of his food for the action of his digestive juices ceases when the former becomes grasped by the muscles of his pharynx. The sandwich is the expedient with which the culinary art meets the demand for a meal in a hurry. By means of it with one bite and some half dozen chews the jaded mortal can convert into a tasty pulp and bolt an adequate quantity of all three typical elements of his diet, and, moreover, can talk the while without running any serious risks of at least an unæsthetic and possibly a dangerous choke. Nowadays one seems never to do simply one thing at a time, and the axiom of the nursery never to talk when you were eating has been absolutely repealed by the usages of modern society. To-day meals are the reverse of silent, and one must be prepared to speak or to laugh, or perhaps to cry, during mastication, and the one expedient which one possesses and uses to enable one to perform these social and masticatory feats is immediate swallowing. The contents of one's mouth, whatever its consistency, is propelled at a second's notice down one's gullet to enable one to give vent to a lively repartee or to chuckle at the repartee of others. The sandwich and its immediate relations, the pâté, the purée, the quenelle, and the mousse are friends in need in this gastronomico-social struggle. In the case of these foods nearly all that culinary mechanics can do has been done; all possible irritating or indigestible constituents of the meat have been carefully removed, the meat fibres themselves have been pounded and ruptured, delicate carminative flavourings with a due proportion of salt have been added, and practically all the consumer has to do is to swallow.

The pâtés and potted meats have, however, also a very serious value and are exceedingly useful in invalid dietetics when one is beginning to put a patient who has been suffering from intestinal irritation upon a meat or fish diet. They further have an advantage from the point of view of domestic economy in that they keep, and householders by having a stock of them in the stores, can provide for any sudden increased demand upon the commissariat. We cannot emphasise too strongly that the great use of these preparations is that they will keep not only before being opened, but also a reasonable time afterwards. To effect this latter desideratum three methods are adopted—namely, the paste is cooked or sterilised, its water-content is reduced, and some salt or other preservative is added. These pastes and pâtés, especially the ones made of ham and fish, occupy a peculiar position in the diet of the ordinary person. They are, as a rule, taken in small quantities spread upon bread and butter, and to some extent are comparable to a sauce or a condiment in that they are used to give taste and flavour to bland articles of diet, but differ from condiments in that they themselves possess distinct nutritive value.

### Samples Received.

We have received from Messrs. Lazenby and Sons, Limited, of 18 Trinity Street, London, S.E., a specimen of their potted beef. This beef is of excellent quality and taste, and exceedingly well made. It forms a very even paste, and spreads well upon bread and butter or toast. It is not too highly flavoured, and the taste of good beef is well pronounced. The same firm have supplied a tin of their potted turkey and ham. We regard this product as sound, wholesome, and made from good materials. Messrs. Traynier, Yallop and Company, Limited, Great Yarmouth, have submitted a sample of their ham and tongue paste and of their ham and veal pâté. We regard both these preparations as in every way satisfactory and to be recommended, and have no doubt that considerable care has been exercised both in the choice of the materials used and in their preparations. Hunter's Handy Ham Company have supplied a tin of their Hhamco. This substance is not a paste, but may be regarded as a modified sausage, although not sausage-shaped. It is intended to be eaten in slices, and is exceedingly well adapted for sandwiches. It can be regarded as thoroughly wholesome and nutritious. We have received from Messrs. Noel and Sons, Soho Works, London, S.E., samples of their bloater and salmon and shrimp pastes. These pastes are put up in glass. They have an exceedingly appetising taste, and are thoroughly well and carefully made. They keep well even after they have been opened, and are pre-eminently suitable as a savoury addition to bread or toast and butter, or when suitably prepared as a basis for a hors d'œuvre or savoury, and adapt themselves well for use with salted biscuits, grated egg yolk, olives, etc. The same have firm sent us a tin of their chicken and ham pâté. This preparation is well up to the standard of the other articles supplied by this firm. Messrs. Tebbutt and Company, Melton Mowbray, have forwarded a specimen of their ham and tongue paste. This paste is put up in glass, and is well adapted for sandwiches or for use at breakfast. It spreads well, and has a good flavour of ham and tongue, and we have no doubt that, as in the case of the other preparations of this firm, the best materials are used for its manufacture. We have received a sample of St. Ivel salmon and shrimp paste prepared by Aplin, Barrett and Company, Limited, Yeovil, Somerset. This paste has a good taste of salmon and shrimp, is put up in glass, and keeps well. It is not too salt, is soft, and of good consistency for spreading; both the shrimps and the salmon are well pounded. As far as we have been able to ascertain, it is a wholesome and desirable product. Messrs. Poulton and Noel, Belgravia Works, London, the well-known manufacturers, have sent us a sample of their potted veal and ham. This preparation is put up in glass, and is exceedingly well made and flavoured. We think it is a most desirable product, and well suited for invalid use. The same firm have also sent us a tin of their potted chicken and ham, which we can also recommend, both with regard to the soundness of the materials used and the method of preparation.

It will be observed that a very large number of preserved prepared meats and pastes have been brought under our inspection by the manufacturers, and it is gratifying to be able to observe that carefully-selected materials and scientific preparation have, in every case investigated by us, had its reward in the production of an article of dietetic value and wholesomeness.

